# Prognostic Impact of Long-term Postoperative Pneumonia in Elderly Patients with Early Gastric Cancer

**DOI:** 10.7150/jca.71349

**Published:** 2022-07-11

**Authors:** Ayako Kamiya, Tsutomu Hayashi, Ryota Sakon, Kenichi Ishizu, Takeyuki Wada, Sho Otsuki, Yukinori Yamagata, Hitoshi Katai, Takaki Yoshikawa

**Affiliations:** Department of Gastric Surgery, National Cancer Center Hospital, Tokyo, Japan.; Ayako Kamiya and Tsutomu Hayashi contributed equally.

**Keywords:** pneumonia, gastrectomy, elderly patients

## Abstract

**Background:** Elderly patients with stage I gastric cancer, a disease that is curable by surgery, easily develop long-term postoperative pneumonia (LTPP) within two years after surgery despite showing no active symptoms. The present study assessed whether or not LTPP determines the later prognosis among elderly patients.

**Methods:** We retrospectively examined patients >75 years old who underwent R0 gastrectomy for gastric cancer and were diagnosed with T1 disease at National Cancer Center Hospital between 2005 and 2012. LTPP was evaluated by chest computed tomography every year until two years after surgery.

**Results:** Of the 3412 patients who underwent gastrectomy in our hospital during this period, 159 were included in this study. The elderly patients who developed LTPP had a worse prognosis than those who did not. Furthermore, their comorbidities and LTPP were significant independent risk factors for a poor prognosis. Patients who developed LTPP had a significantly higher risk of dying due to respiratory disease or cardiovascular disease than those without pneumonia.

**Conclusions**: LTPP was significantly related to a poor survival and death from respiratory disease. To improve the prognosis, not only nutritional support but also exercise and rehabilitation program may be required for patients who develop LTPP within two years after surgery.

## Introduction

Japanese gastric cancer is characterized by early disease and elderly patients 1,2. Early gastric cancer is typically curable with local treatment. The overall survival rate after surgery was reportedly more than 95% among non-elderly patients 3. However, it did not reach 80% in elderly patients over 80 years old 3, possibly due to the high proportion of causes of death other than gastric cancer itself 4. Because gastrectomy easily induces body weight loss, muscle loss, reflux, and aspiration 5,6, elderly patients are at a high risk of pneumonia even if they survive the perioperative period. Pneumonia after gastrectomy is a serious threat in elderly patients, but this has not been explored in detail thus far.

We previously examined pneumonia shadow on chest computed tomography (CT) performed as oncological follow-up in elderly patients who received surgery and were diagnosed with early gastric cancer (submitted for publication). Surprisingly, pneumonia shadow was detected in approximately one fourth of the elderly patients by two years after surgery. To differentiate this entity from perioperative pneumonia, we called it “long-term postoperative pneumonia (LTPP)”. CT detected “LTPP”, in patients who had not shown any active symptoms of pneumonia. Thus, LTPP is a silent shadow of past pneumonia including community-acquired pneumonia after gastrectomy, possibly related to poor daily activity, muscle depletion, reflux, and aspiration. Given the above, we suspected that such elderly patients might be at a high risk of other causes of death, including pneumonia.

To clarify whether or not LTPP occurring within two years after surgery determines the later prognosis in elderly patients, we conducted a follow-up study using the cohort we examined in the previous study (submitted for publication).

## Materials and Methods

### Patients

Patients were selected from the clinical database of consecutive patients who underwent gastrectomy for gastric cancer at National Cancer Center Hospital from January 2005 to December 2012, based on the following criteria: (1) a pathological diagnosis of T1, (2) age ≥75 years old, (3) R0 resection achieved, and (4) chest CT performed before surgery and within two years after surgery. Those who were censored within two years after surgery were excluded.

### Surgery and follow-up

Surgery was basically determined by the Japanese Gastric Cancer Treatment Guideline version 2 or version 3 depending on the date of the surgery 7,8. In short, gastrectomy of D1 or D1+ dissection was performed for early cancer without adjuvant chemotherapy. The surgical procedure and extent of lymphadenectomy were determined by the oncological tumor characteristics, regardless of the patient's age. The postoperative follow-up was as follows: disease recurrence for stage I tumors was evaluated routinely every six months during the first year and every year thereafter for the next four years. The oncological follow-up included physical examinations, blood tests, and CT or ultrasonography. Basically, chest to abdominal CT was performed routinely every year for at least five years after surgery for oncological follow-up. When recurrence was suspected, additional imaging studies were performed.

### Diagnostic criteria for long-term postoperative pneumonia

**Figure 1:**
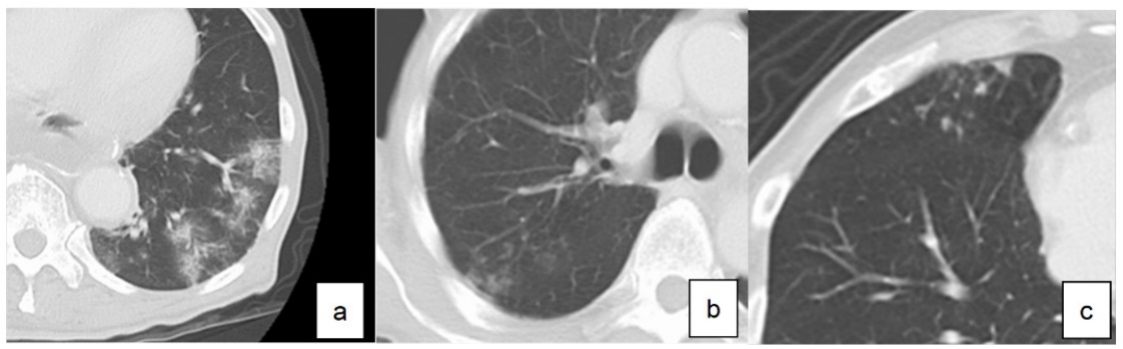
Definition of diagnosis of pneumonia based on computed tomography. **a.** consolidation type, **b.** reticular type, **c.** nodular type

LTPP was diagnosed based on chest CT performed every year until two years after surgery. According to the guidelines for diagnostic imaging of adult community-acquired pneumonia 2007, diagnostic findings of pneumonia on CT were classified into three types (Figure 1): consolidation type, reticular type and nodular type 9. Those presenting two or more types at the same time were called as mixed types. The diagnosis of LTPP was defined as the new presence of these findings on chest CT compared to CT performed before surgery.

After the certificated radiologists checked the radiological findings, two surgeons evaluated the images for the diagnosis of LTPP. If the diagnosis of the two surgeons differed, the images were evaluated again and final judgments were made. The patients were classified into those without LTPP (C group) and those with LTPP (P group).

### Statistical analyses

The SPSS software program, version 15.0 (Statistical Package for the Social Sciences; SPSS, Chicago, IL, USA) was used to perform the statistical calculations. Statistical comparisons of the differences in the age, body mass index (BMI= body weight [kg]/height [m]^2^), Geriatric Nutritional Risk Index (GNRI=14.89×Alb [g/dl]+41.7×(body weight [kg]/ ideal body weight [kg])), Charlson score 10, % vital capacity (%VC) and forced expiratory volume in 1 second (FEV1%) were analyzed by Student's t-test, and other variables were analyzed by the chi-squared test.

In this study, the overall survival (OS) was defined as the period from two years after surgery until the date of the final observation or death. The OS curves were calculated based on the Kaplan-Meier curves and were compared by the log-rank test. The data for patients who did not experience an event by the date of the final observation were treated as censored cases.

Univariate and multivariate Cox proportional hazards regression models were used to analyze the hazard ratios (HRs) for the OS. P values <0.05 were considered to indicate statistical significance. The cumulative incidence rate of death of respiratory diseases and that of cardiovascular diseases between the two groups was compared by Gray's test.

## Results

### Patients' demographics

**Figure 2:**
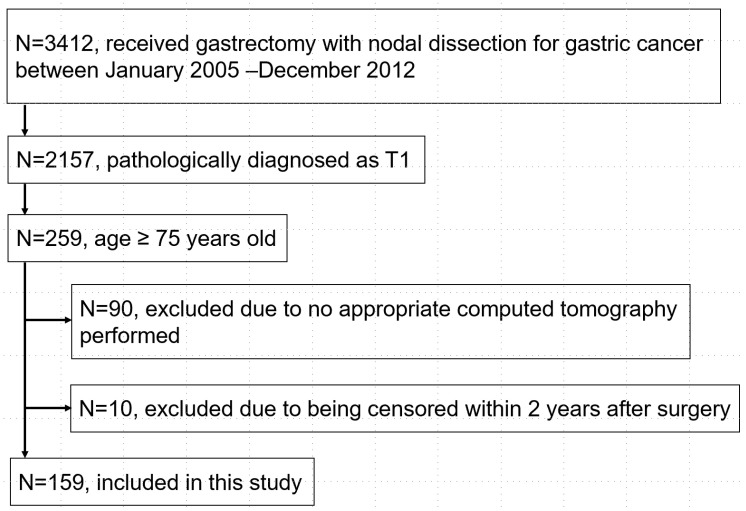
Flow diagram of the present study

Among the 3412 patients who underwent gastrectomy for gastric cancer at National Cancer Center Hospital between 2005 and 2012, 169 had been examined in our previous study (submitted for publication). Figure 2 shows the consort diagram of the present study. Ten cases were censored within two years after surgery and five of them died of other diseases. Thus, a total of 159 patients were included in the present study. LTPP was found in 40 patients (25.2%) by chest CT: consolidation type (n=14), reticular type (n=9), nodular type (n=14), and mixed type (n=3). The agreement rate in the diagnosis of LTPP was 96% in two surgeons.

Table 1 summarizes the clinicopathological characteristics of the patients. All patients were diagnosed as pathological Stage I. Compared with the patients who did not develop LTPP, the proportion of men was significantly higher in those who had LTPP (p=0.025). Other background characteristics including surgical procedure, preoperative respiratory functions, and postoperative complications were similar between the groups. Seventy-two patients had a pneumonia shadow before surgery. Among them, 22 developed LTPP at another location of the lung within 2 years after surgery.

### Survival outcomes

**Figure 3:**
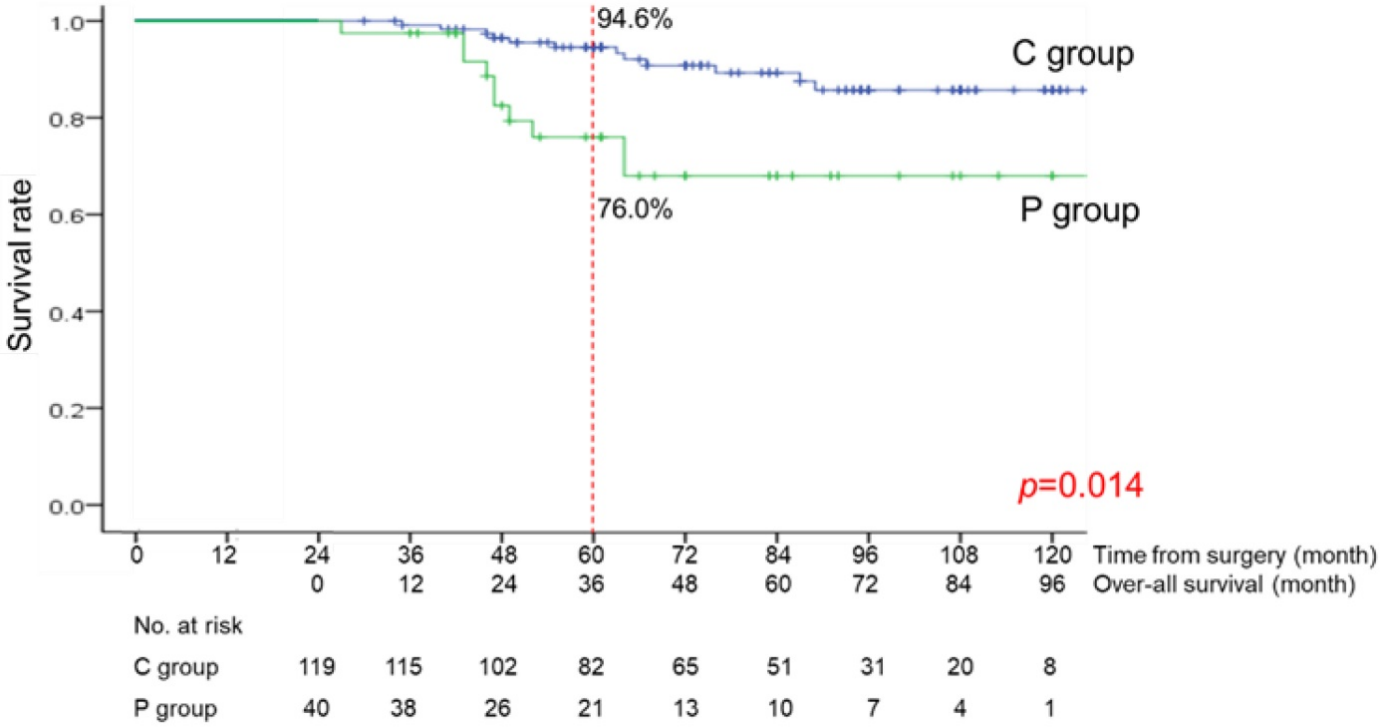
Over-all survival curve of the C group (patients without long-term postoperative pneumonia) and the P group (patients with long-term postoperative pneumonia)

The median follow-up period from the date at 2 years after surgery was 48 months (range, 3-121 months). The OS curves are shown in Figure 3. There was a significant difference between the C and P groups (p=0.014). The OS rate at 3 years was 94.6% in the C group and 76.0% in the P group.

### Prognostic factors predicting the long-term survival

In the univariate analyses, the Charlson score and LTPP were found to be significantly associated with the OS (Table 2). According to the multivariate analysis, significant independent risk factors for the OS were the Charlson score (hazard ratio (HR): 4.057, 95% confidence interval (CI): 1.440-11.43) and LTPP (HR: 2.541, 95% CI: 1.120-5.767) (Table 2).

### Cause of death

**Figure 4:**
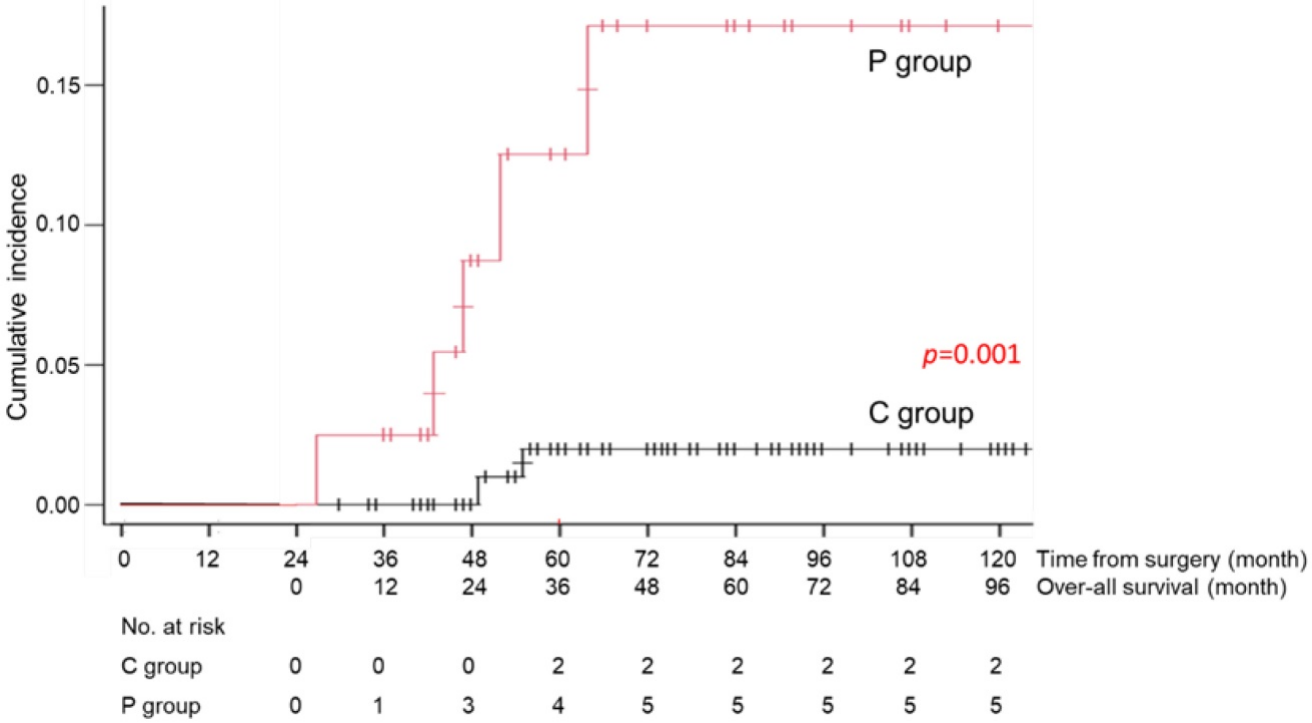
Cumulative incidence rate of death due to respiratory diseases in the C group (patients without long-term postoperative pneumonia) and the P group (patients with long-term postoperative pneumonia)

**Figure 5:**
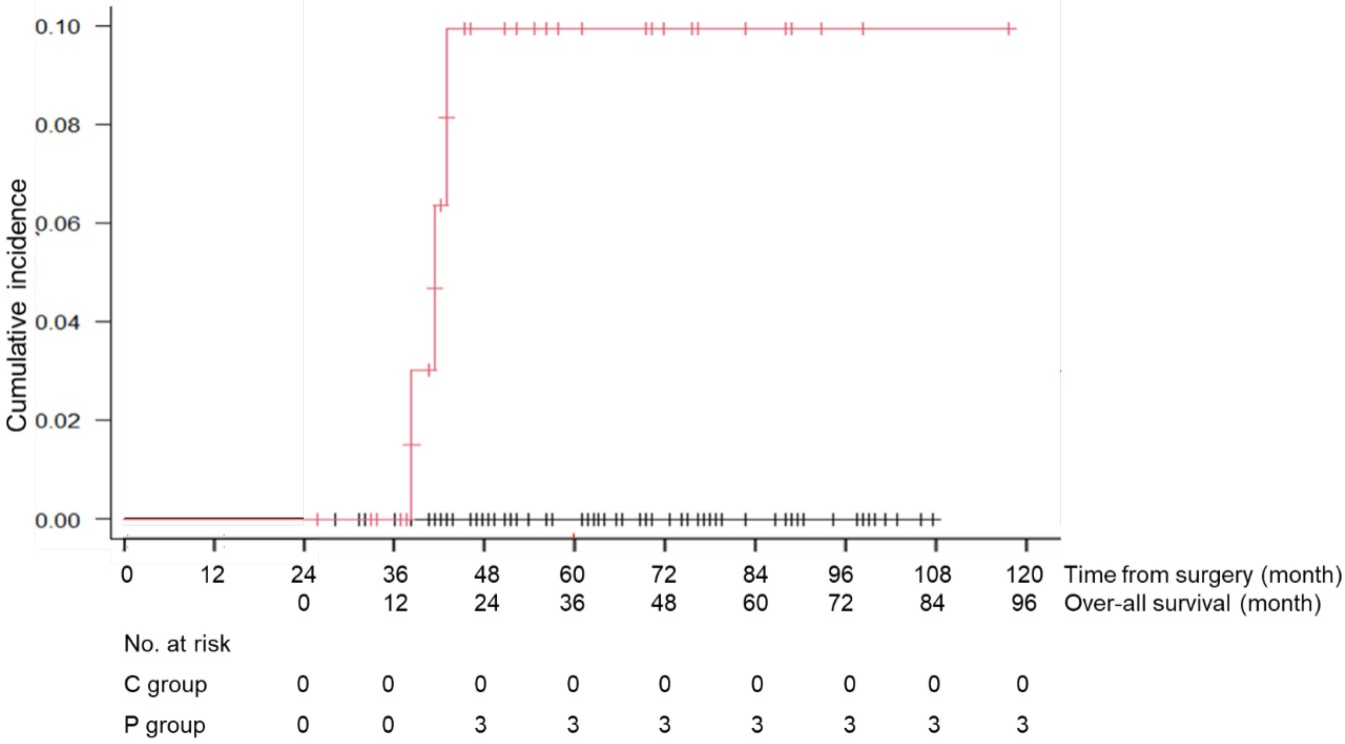
Cumulative incidence rate of death due to cardiovascular diseases in the C group (patients without long-term postoperative pneumonia) and the P group (patients with long-term postoperative pneumonia)

During the follow-up period, 1 patient in the C group died of recurrent disease, and 23 patients (13 patients of the C and 10 patients of the P group) died of causes other than gastric cancer (Table 3). Death due to respiratory disease was more frequently found in the P group than that of the C group (13% vs 2%, p=0.012) and that due to cardiovascular disease was also significantly higher in the P group than that of the C group (8% vs 0%, p=0.015). Other causes of death were similarly low in the both groups. The cumulative incidence rates of death due to respiratory diseases and cardiovascular disease are shown in Figures 4 and 5. There was a significant difference in this rate between the C and P groups (p=0.001 for respiratory diseases and p=0.001 for cardiovascular disease).

## Discussions

We confirmed that approximately one in four elderly patients was diagnosed with LTPP after gastrectomy, that showed much higher incidence in comparison to general elderly people. We demonstrated for the first time that elderly patients who developed LTPP had a worse prognosis than those who did not. Furthermore, comorbidities and LTPP were significant independent risk factors for a poor prognosis. Previous studies have shown that the Charlson comorbidity score was significantly related to a poor prognosis, which was concordant with the present findings 11,12. We found some previous reports that focused on the perioperative pneumonia following gastrectomy as a risk factor for a poor prognosis 13-15. However, there have been no reports concerning the prognostic effect of LTPP.

In the C group, the survival rate at 5 years after surgery was 94.6%, which was as good a prognosis as that in non-elderly general patients with stage I gastric cancer 3. This means that elderly patients after gastrectomy for early gastric cancer will have a good prognosis if they do not develop LTPP within two years after surgery. In contrast, the survival rate at 5 years after surgery in the P group was 76.0%, which was much worse than that in non-elderly patients with early gastric cancer. Thus, LTPP occurring within two years after surgery determined the later prognosis in elderly patients. LTPP might be a surrogate marker for frailty, and more research would be needed on the relationship between LTPP and frailty.

In this study, patients who developed LTPP within two years after surgery had a significantly higher risk of death due to respiratory disease than those who did not. The incidence of death due to respiratory disease was over 10% in the P group but under 5% in the C group. Several previous reports have focused on the survival outcomes and death from other diseases in elderly patients with gastric cancer, particularly in the early stage 16,17. Fujiya et al. reported that 14% of patients died due to other malignancies and 3% died due to respiratory disease 18. Sasako et al. reported that 34% of patients died due to other malignancies and 6% died due to respiratory disease 4. In the present study, we identified patients with a high risk of dying from respiratory disease within two years after gastrectomy. It would not be surprising to see that the patients who developed LTPP within two years after surgery had a high risk of death due to the respiratory disease. LTPP might silently damage the respiratory function due to local inflammatory reactions, resulting in more severe respiratory disease. The difference in the degree of respiratory damage between the two groups would explain the survival difference. To improve the prognosis, we should consider enacting a special respiratory rehabilitation program by selecting patients who develop LTPP within two years after surgery.

Interestingly, death due to cardiovascular disease was also increased in the patients who developed LTPP. It is well known finding that chronic obstructive pulmonary disease induces cardiac failure so called pulmonary heart. In this study, however, LTPP was not related with preoperative respiratory function. On the other hand, previous reports showed that cardiovascular events are frequent in community-acquired pneumonia, and their occurrence adversely affects outcome 19,20. The mechanism remains to be fully established, but it has been suggested that the inflammatory state in patients affected by community-acquired pneumonia acts to promote platelet activation and thrombosis, and to narrow coronary arteries through vasoconstriction 20. Thus, LTPP after gastrectomy would increase the cardiovascular death.

Several limitations associated with the present study warrant mention. First, this was a retrospective single-center study analyzing 159 elderly patients. However, few studies focusing on elderly patients have investigated the clinical importance of LTPP. Therefore, the accumulation of findings from retrospective studies would be meaningful. Our results should be further validated in a multicenter study with a large sample size. Second, preoperative health status, which could be associated with the development of LTPP, was not included in this study. Because the present study was a retrospective study, we retrieved the data from the medical records. However, the preoperative performance status (PS) was not clearly described or missing in many cases. Third, postoperative frailty could be related with LTPP. To evaluate the frailty before developing LTPP, patients have to receive measurement of the muscle strength or volume several times until 1 year after surgery. To do such evaluation for stage I, prospective interventional study is necessary. Therefore, we could not examine the frailty after surgery in this retrospective study. Fourth, the prognosis of patients with LTPP may have been overestimated because our hospital specializes in oncology and often performs surgery for patients with few comorbidities. In the community hospital setting, where surgeons operate on elderly patients with severe comorbidities, patients might have a poorer prognosis.

In conclusion, LTPP within two years after surgery was significantly related to a poor survival and death due to respiratory disease in elderly patients with stage I gastric cancer. To improve the prognosis, not only nutritional support but also exercise and rehabilitation program, such as one involving respiratory rehabilitation, oral care, and swallowing rehabilitation, may be required for especially male patients, who have high risk for LTPP.

## Ethics Committee Approval and Patient Consent

All study participants provided informed consent, and the study design was approved by an ethics review board.

## Figures and Tables

**Table 1:** Clinicopathological characteristics

Variables	Total N (%)^♱^	C group (n=119) N (%)^♱^	P group (n=40) N (%)^♱^	*p* value^♱♱^
Age (median, range)	77 (75-87)	78 (75-87)	77 (75-85)	0.679
Sex				0.025
male	104 (65)	72 (61)	32 (80)	
female	55 (35)	47 (39)	8 (20)	
Surgical approach				0.265
Open	145 (91)	107 (90)	38 (95)	
Laparoscopy	14 (9)	12 (10)	2 (5)	
Surgical procedure				0.991
Distal gastrectomy	75 (47)	55 (46)	20 (50)	
Pylorus preserving gastrectomy	44 (28)	33 (28)	11 (28)	
Total gastrectomy	27 (17)	21 (18)	6 (15)	
Proximal gastrectomy	13 (8)	10 (8)	3 (7)	
BMI (median, range)*	22.6 (16.5-32.2)	22.6 (16.7-32.2)	22.8 (16.5-30.5)	0.787
GNRI (median, range)**	104.5 (89.0-128.0)	104.5 (89.0-125.3)	104.7 (89.9-128.0)	0.861
Charlson Score				0.622
0	88 (55)	69 (58)	19 (48)	
1	34 (22)	24 (20)	10 (25)	
2	23 (14)	15 (12)	8 (20)	
3	8 (5)	6 (5)	2(5)	
4	2 (1)	2 (2)	0	
5	0	0	0	
6	3 (2)	2 (2)	1 (2)	
7	1 (1)	1 (1)	0	
Current smoking history				0.813
+	69 (43)	51 (43)	18 (45)	
-	90 (57)	68 (57)	22 (55)	
%VC (median, range)***	103 (53-175)	108 (53-175)	100 (69-133)	0.697
FEV1% (median, range)****	73 (31-96)	76 (34-92)	71.5 (31-96)	0.900
Sliding Hernia				0.790
+	49 (31)	36 (30)	13 (33)	
-	110 (69)	83 (70)	27 (67)	
Preoperative pneumonia shadow				0.154
+	72 (45)	50 (42)	22 (55)	
-	87 (55)	69 (58)	18 (45)	
Postoperative complications				0.813
+	29 (18)	21 (18)	8 (20)	
-	130 (82)	98 (82)	32 (80)	
Short-term postoperative pneumonia				0.642
+	6 (4)	4 (3)	2 (5)	
-	153 (96)	115 (97)	38 (95)	
Pathological TNM stage				0.454
T1aN0	65 (41)	48 (40)	17 (43)	
T1aN1	1 (1)	0 (0)	1 (2)	
T1bN0	82 (51)	62 (52)	20 (50)	
T1bN1	11 (7)	9 (8)	2 (5)	

^♱^: Number and percentage of patients in each category in each group (except age, BMI, GNRI, %VC and FEV1%) ^♱♱^: Age, BMI, GNRI, %VC and FEV1% were analyzed by Student's t-test, and other variables were analyzed by the chi-square test. BMI*: body mass index =body weight [kg]/ height [m]^2^; GNRI**: Geriatric Nutritional Risk Index =14.89×Alb [g/dl] +41.7× (body weight [kg]/ideal body weight [kg]); %VC***: vital capacity; FEV1%****: forced expiratory volume in 1 second

**Table 2:** Prognostic factors in univariate and multivariate Cox proportional-hazards regression models

Variables	Total N (%)^♱^	Univariate analysis	Multivariate analysis		
		*p* ^♱♱^	Hazard ratio	*p* ^♱♱^	95% confidence interval
Age		0.245			
<80	113 (71)				
80≤	46 (29)				
Sex		0.149	2.021	0.153	0.769-5.310
male	104 (65)				
female	55 (35)				
BMI*		0.446			
<22.5	77 (48)				
22.5≤	82 (52)				
Charlson score		0.007	4.057	0.008	1.440-11.43
<3	145 (91)				
3≤	14 (9)				
Current Smoking History		0.343			
+	69 (43)				
-	90 (57)				
Preoperative pneumonia		0.669			
+	72 (45)				
-	87 (55)				
Postoperative complication		0.175	1.731	0.264	0.661-4.537
+	29 (18)				
-	130 (82)				
Pathological depth of invasion		0.335			
T1a	66				
T1b	93				
LTPP**		0.014	2.541	0.027	1.120-5.767
+	40 (25)				
-	119 (75)				

^♱^: The number and percentage of patients in each category in each group ^♱♱^: Variables were analyzed by the uni- and multivariate Cox proportional hazards regression models. BMI*: Body Mass Index= body weight [kg]/ height [m]^2^; LTPP**: Long-term postoperative pneumonia

**Table 3:** Causes of death of the elderly after gastrectomy for stage I gastric cancer

	Total (n=24) N (%)^♱^	C group (n=14) N (%)^♱^	P group (n=10) N (%)^♱^
Gastric cancer	1 (1)	1 (1)	0 (0)
Respiratory disease	7 (5)	2 (2)	5 (13)
Other malignancy	5 (3)	4 (3)	1 (3)
Cerebrovascular disease	3 (2)	2 (2)	1 (3)
Cardiovascular disease	3 (2)	0 (0)	3 (8)
Intestinal disease	1 (1)	1 (1)	0 (0)
Neurological disease	1 (1)	1 (1)	0 (0)
Senility	1 (1)	1 (1)	0 (0)
Unknown	2 (1)	2 (2)	0 (0)

^♱^: The number and percentage of patients in each category in each group
